# Dr. J. D. Flagg

**Published:** 1889-06

**Authors:** 


Dr. J. D. Flagg, Demonstrator of Anatomy, Medical Department, Niagara University, has removed from No. 98 South Division street to No. 125 East Eagle street.

